# Supplementary material for: The causal role of circulating inflammatory markers in osteoporosis: a bidirectional Mendelian randomized study
Source: Front Immunol. 2024 Jul 18;15:1412298. doi: 10.3389/fimmu.2024.1412298 (PMC11291241; doi:10.3389/fimmu.2024.1412298)
Supplement: Supplementary file 1 [file DataSheet_1.zip › supplementary material/OSTEOPOROSIS-CIM/GCST90274777.tsv.gz_forest.pdf]

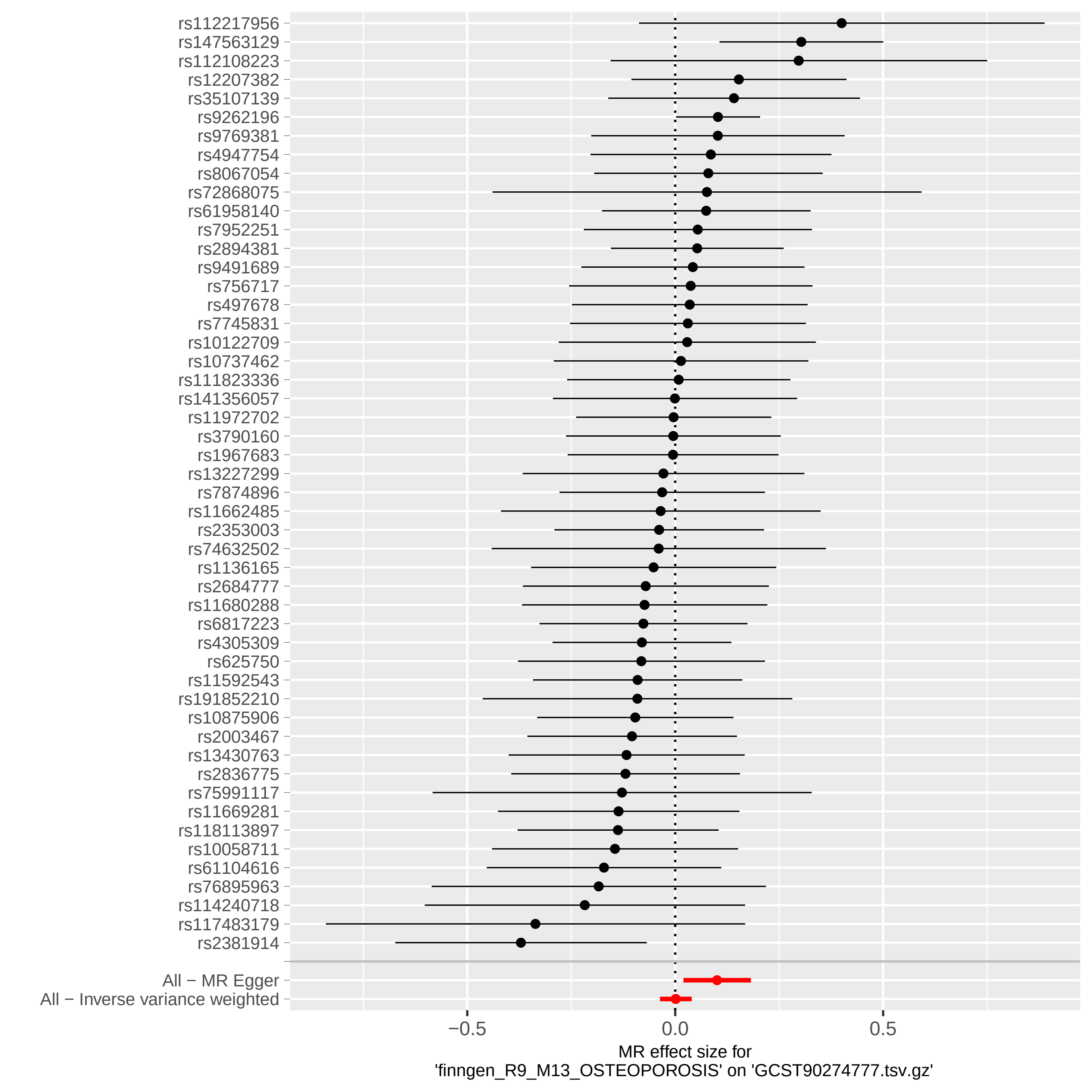
rs112217956
rs147563129
rs112108223
rs12207382
rs35107139
rs9262196
rs9769381
rs4947754
rs8067054
rs72868075
rs61958140
rs7952251
rs2894381
rs9491689
rs756717
rs497678
rs7745831
rs10122709
rs10737462
rs111823336
rs141356057
rs11972702
rs3790160
rs1967683
rs13227299
rs7874896
rs11662485
rs2353003
rs74632502
rs1136165
rs2684777
rs11680288
rs6817223
rs4305309
rs625750
rs11592543
rs191852210
rs10875906
rs2003467
rs13430763
rs2836775
rs75991117
rs11669281
rs118113897
rs10058711
rs61104616
rs76895963
rs114240718
rs117483179
rs2381914
All − MR Egger
All − Inverse variance weighted
−0.5
0.0
0.5
MR effect size for
'finngen_R9_M13_OSTEOPOROSIS' on 'GCST90274777.tsv.gz'
